# Relationship between Brain Age-Related Reduction in Gray Matter and Educational Attainment

**DOI:** 10.1371/journal.pone.0140945

**Published:** 2015-10-16

**Authors:** Patricia Rzezak, Paula Squarzoni, Fabio L. Duran, Tania de Toledo Ferraz Alves, Jaqueline Tamashiro-Duran, Cassio M. Bottino, Salma Ribeiz, Paulo A. Lotufo, Paulo R. Menezes, Marcia Scazufca, Geraldo F. Busatto

**Affiliations:** 1 Laboratory of Psychiatric Neuroimaging, Department and Institute of Psychiatry, Faculty of Medicine, University of São Paulo, São Paulo, São Paulo, Brazil; 2 Center for Interdisciplinary Research on Applied Neurosciences, University of São Paulo, São Paulo, São Paulo, Brazil; 3 Old Age Research Group (PROTER), Department and Institute of Psychiatry, Faculty of Medicine, University of São Paulo, São Paulo, Brazil; 4 Center for Clinical and Epidemiologic Research, University Hospital and Faculty of Medicine, University of São Paulo, São Paulo, Brazil; 5 Department of Preventive Medicine, Faculty of Medicine, University of São Paulo, São Paulo, São Paulo, Brazil; 6 Laboratory of Psychopharmacology and Clinical Psychophysiology (LIM-23), Faculty of Medicine, Institute of Psychiatry, University of São Paulo, São Paulo, São Paulo, Brazil; Brighton and Sussex Medical School, UNITED KINGDOM

## Abstract

Inter-subject variability in age-related brain changes may relate to educational attainment, as suggested by cognitive reserve theories. This voxel-based morphometry study investigated the impact of very low educational level on the relationship between regional gray matter (rGM) volumes and age in healthy elders. Magnetic resonance imaging data were acquired in elders with low educational attainment (less than 4 years) (n = 122) and high educational level (n = 66), pulling together individuals examined using either of three MRI scanners/acquisition protocols. Voxelwise group comparisons showed no rGM differences (p<0.05, family-wise error corrected for multiple comparisons). When within-group voxelwise patterns of linear correlation were compared between high and low education groups, there was one cluster of greater rGM loss with aging in low versus high education elders in the left anterior cingulate cortex (p<0.05, FWE-corrected), as well as a trend in the left dorsomedial prefrontal cortex (p<0.10). These results provide preliminary indication that education might exert subtle protective effects against age-related brain changes in healthy subjects. The anterior cingulate cortex, critical to inhibitory control processes, may be particularly sensitive to such effects, possibly given its involvement in cognitive stimulating activities at school or later throughout life.

## Introduction

Many neuroimaging studies using magnetic resonance imaging (MRI) have demonstrated the presence of morphological changes associated with aging in the living human brain, most often including reductions in regional gray matter (rGM) volumes [1]. Over the course of age-related neurodegenerative disorders such as Alzheimer’s disease (AD), rGM reductions are widely known to occur initially and most prominently in the hippocampus and other limbic medial temporal structures, with subsequent progression to the precuneus, lateral temporal cortex and other neocortical regions [2–4]. Conversely, brain aging in healthy individuals is characterized by progressive gray matter (GM) loss in the frontal cortex and other neocortical regions, with relative preservation of the hippocampal region and other limbic / paralimbic GM regions up until approximately 70 years of age. This has been demonstrated by a large number of cross-sectional MRI studies with representative samples of healthy elders investigating linear correlations between brain volumes and age [5–8], as well as a limited number of longitudinal MRI studies with repeated measurements in the same subjects over time [9,10].

Despite the overall consistency of the above findings, MRI studies evaluating age-related brain changes have often documented a large degree of inter-subject variability of imaging measurements [11,12]. The exact mechanisms underlying such inter-subject variations in age-related brain changes and the factors that most significantly influence such variability have not yet been fully elucidated. In AD, it has been proposed that the degree of inter-subject variability in brain degenerative changes is influenced by the efficiency and evolution of compensatory brain mechanisms over time [13]. The concept of cognitive reserve (CR) was originally proposed to explain the lack of a direct relationship between the degree of brain pathology or brain damage versus the kind and severity of the clinical manifestations that should supposedly result from such damage. CR is the hypothesized capacity of the mature adult brain to compensate for the effects of a disease or injury that would be sufficient to cause clinical dementia in an individual with less CR (Stern, 2002). Applied to AD, this hypothesis predicts that older adults with higher CR will have a lower risk of developing overt features of dementia than individuals with less CR. Educational attainment is seen as one of the most relevant elements of CR [14,15]. Education is related to the acquisition and constant exercising of higher-order cognitive skills such as attention, executive functions, memory and language [16]. Low levels of education have been consistently associated with a higher prevalence of dementia and AD in epidemiological studies [17–19] as well as being a risk factor for an earlier onset of dementia [20]. In agreement with such epidemiological findings, several neuroimaging studies have demonstrated that AD-related brain changes are associated with lesser cognitive impairment in elderly individuals with higher levels of previous education [21–24].

In healthy individuals, the stimulation of cognitive skills is accompanied by increased rGM volumes in the brain (most prominently in frontal and parietal neocortical regions), as demonstrated in MRI studies evaluating the effects of cognitive-training programs [25,26]. However, in contrast with the above MRI findings in groups of AD subjects, it is still not clear how and if education influences on the pattern of age-related rGM reductions typically detected in neuroimaging studies of normal aging populations. Very few morphometric MRI studies investigated associations between brain volumes and education in subjects with no cognitive complaints, with conflicting results: while some have found no differences in brain volume according to educational level [21], others have reported differences in frontal, temporal, parietal, occipital and anterior cingulate regions [14]. Moreover, it is still not clear if healthy elders with very low educational attainment show more pronounced rGM volume reduction over time, in other words, if ageing healthy individuals with very low education are more vulnerable to brain atrophy than elders who were more exposed to a cognitive enriched environment.

The aim of the present cross-sectional study is to verify the impact of very low educational attainment on the relationship between rGM volumes and age in elderly healthy subjects. We hypothesize that, compared to elders with higher levels of education, elders with very low educational attainment (less than 4 years of formal education) will show a more pronounced degree of age-related GM reduction in brain regions known as critical to higher-order cognitive processing, including the prefrontal cortex, anterior cingulate cortex and other neocortical regions.

## Material and Methods

### Participants

The local ethical review board from the Faculty of Medicine of the University of Sao Paulo, approved the study, and all participants provided written informed consent to participate. Three different MRI studies (studies 1, 2 and 3) carried out at the Department and Institute of Psychiatry, University of São Paulo Medical School from 2002 to 2010 [27–29] contributed data to the present investigation.

The present study included 188 dementia free elderly subjects who underwent 1.5-T structural T1-weighted MRI scanning (see Table 1). All participants were free of symptoms suggestive of physical or mental disorders based on a structured psychiatric interview, general medical questioning, physical and neurological examination. Cognitive functioning of all participants was evaluated with detailed neuropsychological evaluations carried out to exclude cases of dementia cases and to discard the presence of cognitive deficits greater than what would be expected for the age range of the samples. Study 1 evaluated patients using the Community Screening Instrument for Dementia (CSI-D) [30], the Geriatric Mental State Examination (GMS) [31], an adapted version of the Consortium to Establish a Registry for Alzheimer’s Disease (CERAD) ten-word list learning task with delayed recall, and the animal naming verbal fluency task from the CERAD [32]. Study 2 applied the Mini-mental State Examination (MMSE) [33], sub-items for language comprehension, praxis, remote memory and recent memory from the Cambridge Mental Disorders of the Elderly Examination (CAMDEX) section for assessment of cognitive functions (CAMCOG) [34], a category fluency test [35], the Fuld Object-Memory Evaluation (FOME) [36] and an auditory verbal learning test. Finally, study 3 used the MMSE and the CAMCOG (for further methodological details of neuropsychological assessments see Busatto et al. [29], Ribeiz et al. [28] and Squarzoni et al. [37]).

**Table 1:** Demographic characteristics of the participants according to their educational level.

	High Education (n = 66)	Low Education (n = 122)	Statistical Test	
	**Mean (SD)**	**Mean (SD)**	**t**	**p**
Age (years)	69.58 (4.33)	70.63 (2.93)	1.98	0.049*
Years of education	9.91 (3.73)	2.89 (1.33)	-18.72	<0.001*
	**N (%)**	**N (%)**	**x** ^**2**^	**p**
Male	35 (53.0%)	55 (45.1%)	1.08	0.298

* Refers to statistical significant differences.

Other exclusion criteria were: age under 60 or over 85 years; a current or previous history of neuropsychiatric disorders; mental retardation; severe general medical conditions; missing clinical data; presence of contra-indications for MRI scanning; and presence of silent gross brain abnormalities or artifacts during MRI scanning.

Participants were either recruited through local advertisements or selected from a community-based database of elderly individuals recruited for the São Paulo Ageing & Health (SPAH) study [38], an epidemiological investigation aimed at determining the incidence and prevalence of dementia, other mental disorders, and risk factors for these conditions. The characteristics of the SPAH study have been described in detail elsewhere [38]. In brief, all residents aged 65 or above (n = 2,072) of pre-defined census sectors of an economically disadvantaged area of São Paulo were contacted [38,39].

### Education Measurement

Education attainment was measured according to the total years of formal education informed by the participant in an interview, at the time of MRI scanning. In brief, we considered 4 years of education if subjects completed the fourth grade (i.e. primary school), 8 years if subjects completed the eighth grade (i.e. secondary school), 11 years if subjects completed high school, and 15 years or more if subjects completed college. The sum of years dedicated to post-graduation was added to the final sum. When one of these educational periods was not completed, the number of years until dropout was considered in the estimate of mean years of education. Participants were categorized into low education if they had four or less years of formal education and as high education if they had more than four years of schooling.

## Data Acquisition and MRI Processing

The parameters for acquisition of the structural MRI scans are listed in Table A in S1 File in the data supplement. After extensive quality checking, 188 participants (66 participants with high education and 122 participants with low education; Table 1) were included in the analysis.

Imaging data processing was carried out using optimized voxel-based morphometry (VBM) with the Statistical Parametric Mapping software (SPM8; Wellcome Department of Imaging Neuroscience, London, UK), executed in Matlab Version r2012a (MathWorks, Natick, Mass). First, all anatomical images were reoriented; the mm coordinate of the anterior commissure matched the origin xyz (0, 0, 0), and the orientation approximated Montreal Neurological Institute (MNI) space. Then, all images were segmented and classified into GM, white matter (WM) and cerebrospinal fluid (CSF) using the unified segmentation implemented in SPM8, which provides both the native space versions and Diffeomorphic Anatomical Registration using Exponentiated Lie algebra (DARTEL) imported versions of the tissues [40]. A customized template was created from the subjects using the DARTEL protocol [41,42]. The deformation field was applied to the segmented images in sequence. Finally, the images created in the previous step were standardized to MNI space, re-sliced to 1.5x1.5x1.5mm voxels and smoothed using an 8-mm full width at half maximum (FWHM) Gaussian kernel. The total GM volumes were obtained from the modulated images. The choice to use a 8 mm Gaussian filtering size in the current study was based on the fact that such degree of smoothing provides an optimal degree of increment in signal-to-noise ratio and conformation of MRI data to a normal distribution (thus allowing the use of parametric tests in subsequent statistical comparisons), as well as compensating for some of the data loss incurred by spatial normalization [43,44].

### Statistical Analysis

Descriptive analyses and group comparisons for demographic data were carried out using the Statistical Package for Social Sciences (SPSS) for Windows (version 17—IBM, Armonk, N.Y.). The significance threshold was set at 0.05, two-tailed.

For the brain imaging measurements, we initially carried out between-group (high and low educational level) whole-brain voxelwise comparisons using an analysis of covariance (ANCOVA) model in SPM8, considering age, gender and scanner protocol as confounding factors. Subsequently, with the aim of verifying the impact of education on the relationship between GM volumes and age, we included “group status” as a predictor variable in a voxelwise analysis investigating the presence of significant correlations between GM volumes and age across the High and Low education groups, searching for voxels where there were significant differences in the pattern of rGM volume-age linear correlations between groups. Gender and scanner protocol were considered nuisance factors.

Only voxels with values above an absolute GM threshold of 0.05 entered each of the above analyses. Resulting statistics were thresholded at a p<0.001 level of significance (Z = 3.09) and displayed as SPMs into standard anatomical space. Initially, the SPMs were inspected in order to identify the presence of significant (i) differences in rGM volumes, and (ii) correlations between rGM volumes and age in each of the two groups across the entire brain. Findings in this exploratory inspection were reported as significant only if they retained significance after using family-wise error (FWE) correction for multiple comparisons over the entire brain (p<0.05). Subsequently, the SPMs were inspected on a hypothesis-driven fashion in order to search for significant clusters of voxels in brain regions where significant correlations/differences had been predicted a priori (i.e. anterior cingulate cortex, dorsal prefrontal cortex, lateral parietal cortex and medial parietal cortex—precuneus [5,8,14,21]. This hypothesis-driven analysis was conducted using the small volume correction (SVC) method, with the purpose of constraining the total number of voxels included in the analysis. Each region-of interest (ROI) was circumscribed by applying spatially normalized volumes onto the SPMs, based on the anatomical volumes of interest available within the Automatic Anatomical Labeling (AAL) SPM toolbox. Findings in these a priori ROIs were reported as significant using an SVC-based, FWE-corrected statistical threshold of p<0.05 and cluster size above 50.

When reporting significant findings of all voxel-based analysis above, we converted the MNI coordinates of voxels with maximal statistical significances using the Tailarach and Tournoux system [45].

All the statistical procedures described above were carried out twice, firstly for the whole dataset from the three different studies that provided subjects to the present research, and secondly including only subjects from Study 1 (n = 156). This approach was conducted in order to minimize the possible confounding impact of the use of different MRI scanners (GE-Signa and Philips-Gyroscan) and different pulse sequences (SPGR, FSGE and FFE T1), with different acquisition parameters, between the 3 studies that contributed data to the present investigation. Also with the aim of estimating the potential confounding influence of differences in scanning parameters between the 3 studies, regression analyses were performed with SPSS 18.0 software in order to investigate if there were differences in regional brain volumes between scanners/protocols (pooling together in each study the individuals with high and low education). Scanner/protocol was the fixed factor and the dependent variables were the estimates of regional GM volumes extracted from the spatially normalized images of each subject (corrected for the global amount of GM in the brain), using each of the ROI masks described above. Age and gender were included in the model as confounding factors.

## Results

### Demographic characteristics of the sample

Although the two groups of subjects (high versus low education) had a similar proportion of male participants (x^2^ = 1.08; p = 0.298), subjects with high education were slightly younger than low education participants (t = 1.98; p = 0.049). (Table 1).

Considering scan sequence/site, subjects examined in each of the three studies that contributed MRI data had on average similar age, and the same proportion of male participants. Nevertheless, subjects from study 1 had on average fewer years of formal education and, consequently, had a higher proportion of participants allocated to the Low education group (F = 14.10; p<0.001) (Table B in S1 File).

Participants with high and low education were similarly distributed across the age ranges in Studies 1 and 2 (which together contributed to 90.4% of subjects to the present investigation), with a similar proportion of individuals aged less than 70 years or aged 70 or above in those two studies (x^2^ = 0.654; p>0.05). In Study 3 (with n = 18), there was a greater proportion of participants with less than 70 years-old in the high educational group (p_fisher_ = 0.036) (Table C in S1 File).

### Group Comparisons of imaging measurements

#### GM volume differences according to educational level

The mean sum of GM voxels (total GM volume) in the segmented GM images totaled a volume of 639.50 ± 55.78 milliliters in the High education group and of 630.54 ± 54.25 milliliters in the Low education group. This difference was not statistically significant (t = -1.07; p = 0.286). Besides, the whole-brain voxelwise exploratory search for significant rGM volume differences between participants with high and low educational level resulted in no significant results after correction for multiple comparisons. The SVC analysis also resulted in no clusters of significant rGM volume differences between the high and low education groups.

For the analyses including only individuals from Study 1, the total GM volume in the segmented GM images totaled a volume of 634.28 ± 53.64 milliliters in the High education group and of 650.73 ± 60.79 milliliters in the Low education group. This difference was not statistically significant (t = -1.66; p = 0.099). The whole-brain voxelwise search for significant rGM volume differences between subjects with high and low educational level resulted in no significant results after correction for multiple comparisons. The SVC analysis also resulted in no cluster of significant rGM volume differences between the two groups.

#### Between-group differences in the pattern of linear correlation between GM volume and age

**Fig 1:**
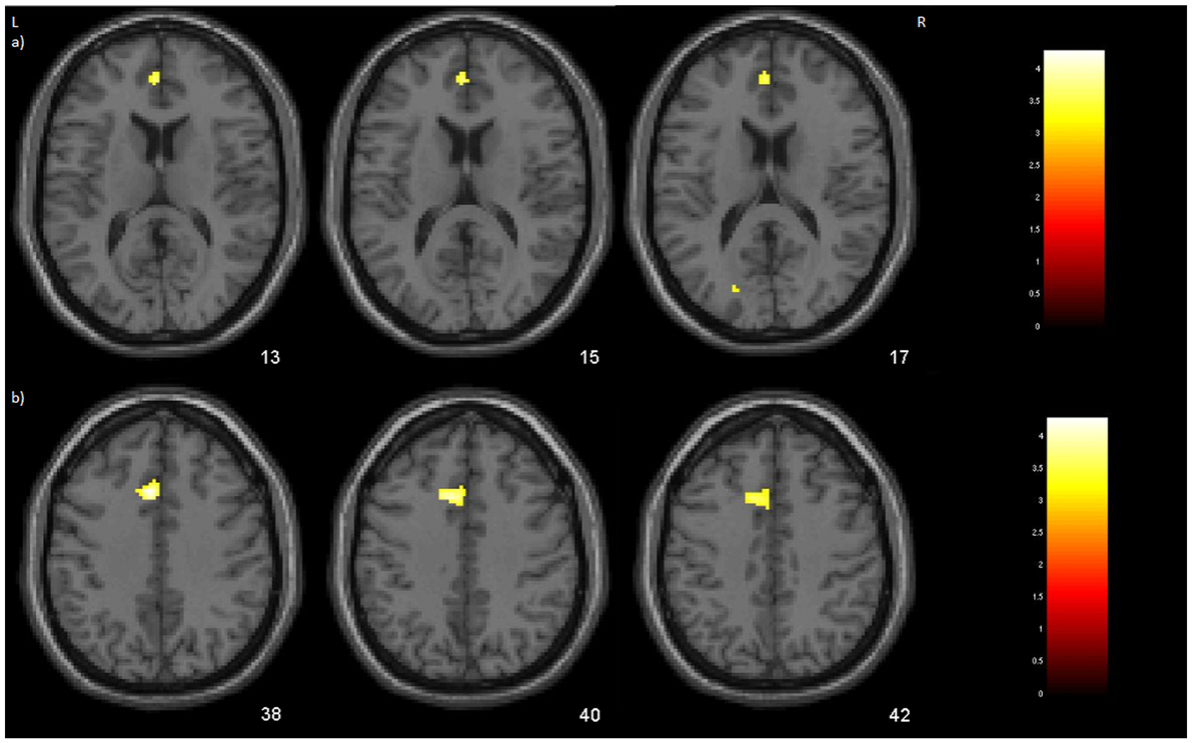
Between-group differences in the pattern of linear correlation between GM volume and age. Results from the inclusion of “group status” as a predictor variable in the analysis investigating within-group correlations between rGM volumes and age across the High and Low education samples. Foci of significance were overlaid on axial brain slices spatially normalized into an approximation to the Tailarach and Tournoux stereotactic atlas (1998). The numbers associated with each frame represent standard coordinates in the z-axis, indicated in MNI (Montreal Neurological Institute). a) One cluster of significant relative loss with aging in low education elders compared with high education subjects was detected in the left anterior cingulate cortex (p<0.05, FWE-corrected for multiple comparisons); b) trend towards more pronounced direct correlation between rGM and aging in the low education than the high education group in the left frontal dorsal region (p<0.10, FWE-corrected). Details for each cluster are provided in Table 2 including coordinates of voxels of maximal statistical significance and their peak Z-score; L = left; R = right.

The inclusion of “group status” as a predictor variable in the analysis investigating correlations between rGM volumes and age across the High and Low education groups revealed no foci of significant interactions in the whole-brain voxelwise analysis, when FWE correction for multiple comparisons was applied. In the SVC analyses, one cluster of significant relative loss with aging in low education elders compared with high education subjects was detected in the left anterior cingulate cortex (Table 2, Fig 1a). A tendency toward significance was observed in the left frontal dorsal region, with the low education group showing a trend towards more pronounced direct correlation between rGM and aging than the high education group (Table 2, Fig 1b).

**Table 2:** Voxelwise differences between subjects with low education (*n* = 122) and high education (*n* = 66) in the significance of linear correlations between regional gray matter volumes and age.

Brain regions (SVC)^a^	Side	Size of Cluster^b^	Peak Z score^c^	Coordinates x,y,z (MNI)^d^	p_FWE_ corrected^e^
*Relative loss with aging in low education elders compared with high education subjects*					
Anterior Cingulate Cortex	L	72	3.63	-2 48 15	0.018*
Dorsal Frontal Cortex	L	68	3.85	-9 18 40	0.069

* Refers to statistical significant differences.

FWE indicates Family-wise error.

^a^ Each region was circumscribed using the small volume correction (SVC) approach, with anatomically defined volume-of-interest masks.

^b^Number of contiguous voxels that surpassed the initial threshold of p<0.001 (uncorrected) in the statistical parametric maps.

^c^Z scores for the voxel of maximal statistical significance.

^d^MNI coordinates of the voxel of maximal statistical significance within each cluster.

^e^Statistical significance after correction for multiple comparisons; inferences made at the level of individual voxels (FWE- correction for multiple comparisons

When we restricted this analysis to a single population of subjects (Study 1) no foci of significant interactions were observed in the whole-brain voxelwise analysis or in the SVC analyses, after the inclusion on “group status” as a predictor variable in the search for an association between rGM volumes and age according do educational level. In order to verify whether there were statistical trends involving the brain regions where significant findings were found in the analyses for the entire sample, we inspected the SPMs using a more flexible threshold of Z = 2.33 (p<0.01, uncorrected), with an extent threshold of 50 voxels. Such inspection of SPMs showed differences between groups in the pattern of intra-group correlation bilaterally in the anterior cingulate cortex (452 voxels, peak coordinates_xyz_ = -29 44 6, z = 3.40, p_uncorr_<0.001 (left); 81 voxels, peak coordinates_xyz_ = 14 42 22, z = 2.81, p_uncorr_ = 0.002 (right)) and the left dorsal frontal cortex (57 voxels, peak coordinates_xyz_ = -14 18 42, z = 2.81, p_uncorr_ = 0.002) in the same locations as reported for the entire sample, as well as bilaterally in the hippocampus (298 voxels, peak coordinates_xyz_ = -18 -7 -14, z = 2.99, p_uncorr_ = 0.002 (left); 188 voxels, peak coordinates_xyz_ = 17 -4 -12, z = 2.87, p_uncorr_ = 0.002 (right)), orbitofrontal cortex (226 voxels, peak coordinates_xyz_ = -11 50 -18, z = 2.90, p_uncorr_ = 0.002 (left); 126 voxels, peak coordinates_xyz_ = 14 54 -18, z = 2.97, p_uncorr_ = 0.001 (right)), and medial parietal cortex (293 voxels, peak coordinates_xyz_ = -11 -87 13, z = 3.00, p_uncorr_ = 0.001 (left); 104 voxels, peak coordinates_xyz_ = 18 -87 -3, z = 3.17, p_uncorr_ = 0.001 (right)).

### Brain volume differences between MRI scanners/sequence parameters

When the relative volume sizes of *a priori* selected brain regions were compared between the 3 studies (pulling together individuals with low and high education) (Table 3), we verified that scanner/sequence had an impact over the volume size of the following ROIs: anterior cingulate cortex (left [F = 20.05; p<0.001] and right [F = 24.61; p<0.001); dorsal frontal cortex (left [F = 3.92; p = 0.022] and right [F = 6.87; p = 0.001]); hippocampus (right [F = 4.78; p = 0.009]); parahippocampal gyrus (left [F = 10.85; p<0.001] and right [F = 7.84; p = 0.001]); and precuneus (left [F = 3.94; p = 0.021] and right [F = 7.62; p = 0.001]). Most of the differences were due to relative smaller brain volumes in Study 1, when compared both to Study 2 (i.e. anterior cingulate cortex, dorsal frontal cortex, parahippocampal gyrus and precuneus) and Study 3 (i.e. anterior cingulate cortex, and parahippocampal gyrus). Participants from Study 3 showed smaller brain volumes than participants from Study 2 for some of the *a priori* ROIs (i.e. anterior cingulate cortex and dorsal frontal cortex) (Table 3).

**Table 3:** Differences in regional GM volumes between scanners / acquisition protocols.

	Study 1 Mean (SD)	Study 2 Mean (SD)	Study 3 Mean (SD)	F	p	Study 1 x 2	Study 1 x 3	Study 2 x 3
Ant Cing L	0.0076 (0.0005)	0.0084 (0.0006)	0.0080 (0.0006)	20.051	<0.001*	<0.001*	0.003*	0.124
Ant Cing R	0.0070 (0.0005)	0.0080 (0.0005)	0.0073 (0.0005)	24.606	<0.001*	<0.001*	0.031*	0.002*
Dors Front L	0.0612 (0.0030)	0.0636 (0.0032)	0.0615 (0.0037)	3.915	0.022*	0.017*	0.998	0.115
Dors Front R	0.0618 (0.0027)	0.0648 (0.0038)	0.0613 (0.0039)	6.867	0.001*	0.002*	0.782	0.003*
Hippoc L	0.0062 (0.0004)	0.0060 (0.0004)	0.0060 (0.0003)	2.739	0.067			
Hippoc R	0.0059 (0.0004)	0.0057 (0.0005)	0.0056 (0.0003)	4.778	0.009*	0.123	0.041*	0.998
Parahippoc L	0.0052 (0.0003)	0.0055 (0.0002)	0.0054 (0.0002)	10.847	<0.001*	0.003*	0.002*	0.997
Parahippoc R	0.0065 (0.0005)	0.0069 (0.0004)	0.0067 (0.0002)	7.836	0.001*	0.004*	0.040*	0.813
Precuneus L	0.0161 (0.0012)	0.0167 (0.0011)	0.0167 (0.0006)	3.941	0.021*	0.177	0.076	0.999
Precuneus R	0.0137 (0.0009)	0.0147 (0.0011)	0.0139 (0.0007)	7.620	0.001*	<0.001*	0.713	0.063

* Refers to statistical significant differences;

L: left; R: right; Ant Cing: Anterior Cingulate Cortex; Dors Front: Dorsal Frontal Cortex; Hippoc: Hippocampus; Parahippoc: Parahippocampus

## Discussion

In the present study, we aimed to verify the impact of education on the relationship between GM volumes and aging in elders with no previous or recent history of neuropsychiatric conditions and with normal cognitive functioning. To the best of our knowledge, this is the first study to investigate the relationship between educational level and brain morphology in an elderly sample including a representative subgroup of participants with four or less years of formal education. Our results suggest that educational level might exert some impact on brain structure in elderly individuals; however, such impact would be restricted to selected, medially located anterior cortical regions and detectable only when considering between-group differences in the linear relationship between regional GM volumes and age.

Strengths of the present study are the inclusion of participants with well-described education attainment, including those with very low levels of education. It is reasonable to assume that a subtle impact of education on age-related brain changes should be more visible when contrasting people with very low (or absent) levels of education to those with longer educational attainment. Previous MRI studies investigating the impact of education on brain morphology have rarely quantified in years the time spent by subjects in schooling, instead simply categorizing the groups of high and low education in respect to educational diploma achieved [14,21]. The findings of the present study provide preliminary evidence suggestive that attending to at least some years of formal education in the infancy might have some impact on brain atrophy several years later.

The concept that education could serve as a protective factor against aging processes in the brain was first developed within the literature of dementia. Several studies demonstrated that patients with AD with higher levels of educational attainment, with similar cognitive symptomatology, endured more brain pathology than their dementing counterparts with lower educational level [21–23]. According to some studies, education could positively impact the effects of aging in two ways. Through a direct pathway, people with higher levels of education may have been exposed to greater cognitive stimulation during school environment. In a more indirect way, education could positively influence the development and maintenance of cognitive abilities throughout life, and such cognitive abilities could in turn lead to healthier behaviors [46]. Both pathways are assumed to potentially halter the development and progression of AD (Kemppainen et al., 2007).

The above assumptions leads to the questioning as to whether the same education-related factors could be protective to people without dementia or other forms of pathological cognitive decline, since healthy elders with higher educational level would also be more cognitively stimulated along their life compared to healthy elders with lower educational attainment. Only a small number of MRI studies of healthy individuals to date have investigated the impact of education on brain volumes using a between-group comparison approach, and they presented divergent data. While Foubert-Samier et al. [14] demonstrated volume reductions in the frontal, temporal, parietal, occipital and anterior cingulate regions in subjects with lower education (participants with primary school with and without a diploma and short secondary school) compared to those with high education (elders with long secondary school and university level), Liu et al. [21] did not find any differences in brain volumes between groups divided according to educational level (more or less than 9 years of education). In the present study, between-group comparisons also resulted in no significant differences in GM volumes between elders with high and low educational experience. Our results were the same both with the total samples and with the subsamples of individuals investigated in Study 1. This indicates that such negative findings were not influenced by differences in MRI scanning parameters.

Methodological differences between studies may be responsible for the above divergent findings. We have strictly selected healthy elders with no signs of any neuropsychiatric disorder as assessed by a semi-structured interview and no cognitive complaints other than those that would be normally expected at their age range, as verified in a comprehensive neuropsychological evaluation. Moreover, the low education group consisted of participants with no more than four years of formal education, with 23 of them being considered illiterate (i.e. no more than one year of schooling). The absence of between-group GM differences in our study provides indication that elders with very low levels of education display no large differences in GM volumes when directly compared to highly educated elders matched for other demographic variables.

Conversely, another way to verify the possible impact of the educational level on brain volumes of healthy elders is by exploring the interaction between brain volume and aging, in other words, by comparing the patterns of within-group correlations between GM volume and age. Our findings provide some indication that people with very low educational achievement may show more GM reduction in the left anterior cingulate cortex as they age than those with high educational level. Despite the novelty of such finding, it is noteworthy that one elegant longitudinal MRI study previously failed to detect significant longitudinal annual atrophy or atrophy–age correlations in the anterior cingulate cortex in a healthy elder sample from the ADNI database [47], thus suggesting that this brain region would be less vulnerable to the effects of aging. Unfortunately, however, information regarding the educational level of the elderly sample in that study was not provided [47], thus precluding direct comparisons between their results and ours.

The roles of the anterior cingulate cortex in cognitive and emotional processing have been widely investigated in recent years. This brain structure has been implicated in several higher-order cognitive functions, including attention or executive functions, monitoring competition, complex motor control, motivation, novelty, error detection and working memory, and anticipation of cognitively demanding tasks [48]. Influential models have emphasized a central role of the anterior cingulate cortex in monitoring conflict as well as in inhibitory control abilities [49–51]. According to this hypothesis, a critical role of the anterior cingulate cortex is to signal conflict to the cognitive control system supported through dorsolateral prefrontal cortices. The cognitive control system will then activate task-relevant information and inhibit task-irrelevant information to resolve the information processing conflict. In this context, it is tempting to suggest that the school environment, by promoting the development of such abilities and the continuous use of efficient cognitive control strategies thereafter, could make the anterior cingulate cortex a brain region less vulnerable to the effects of aging.

Consistent with the above hypothesis, an MRI study with a selected group of healthy persons with 80 years of age or older, identified as “SuperAgers” on the basis of memory test scores, demonstrated that the anterior cingulate cortex was thicker in the SuperAger group even when they were compared to cognitively normal 50- to 65-year-old subjects [52].

The molecular substrate underlying a possible prevention of age-related GM volume loss in the anterior cingulate cortex in healthy elders would need to be further elucidated. Recent studies in non-aged rodents have demonstrated that exposure to an enriched environment enhances the expression of the plasticity gene, egr-1, in the anterior cingulate cortex accompanied by increased long-term potentiation and decreased cingulate long-term depression [53]. To the best of our knowledge, there are no previous studies investigating whether enriched environments also influence neuronal plasticity in the anterior cingulate cortex in aged rodents.

In addition to the significant finding in the anterior cingulate cortex discussed above, we also detected a significant trend difference in the linear relationship between regional GM volumes and age across high and low education groups in the dorsal frontal region. This result helps to raise one other possible explanation for our between-group differences in GM-age correlations, implicating prefrontal brain areas in age-related compensatory over-recruitment. Functional MRI studies have consistently demonstrated that older adults activate some brain regions to a greater degree than young adults during performance of higher-order cognitive operations [54,55]. Thus, several studies have focused on the prefrontal cortex as the primary site of age-related over-recruitment, and have suggested that over-recruitment may reflect attempted compensation for declining functioning in other regions in the aging brain [56]. It has been previously proposed that educational experience may interfere with the compensation abilities of the mature brain during aging [57]. Although caution must be exercised when extrapolating such functional MRI findings related to brain activity to explain morphological changes [58], it is tempting to speculate that such compensation abilities would influence on lesser frontal dorsal brain volume decline with aging in more educated elderly individuals, as suggested by our findings.

Although our study design afforded greater statistical power by retrospectively including a relatively large sample of 188 healthy subjects with no previous or actual history of neuropsychiatric disorders or significant cognitive complaints, such experimental gain could be potentially offset by the introduction of possible confounding factors, such as differences in MRI scanner hardware / imaging sequence. There is evidence that MRI acquisitions with different head coils, different pulse sequences and different voxel dimensions can add significant variability to brain imaging measurements [59]. Although we included in our statistical modelling site/scanning sequence as a confounding factor, we cannot rule out the possibility that differences in scanning parameters would have increased the risk of false-positive results. Actually, when we repeated the analyses using datasets only from Study 1, the significant differences in the relationships between aging and GM volumes in the anterior cingulate and prefrontal cortices were only detectable at a very flexible voxelwise threshold of p<0.01, uncorrected for multiple comparisons. Moreover, when the relative volume sizes of *a priori* selected brain regions were compared between the 3 studies (pulling together individuals with low and high education), we observed smaller brain volume sizes in participants from Study 1, and such brain volume differences were most prominent in the anterior cingulate cortex (although this pattern might also have been influenced by the larger number of low-educated individuals in Study 1). Overall, the interpretation of our findings of possible effects of education on the interaction between aging and GM volumes of the prefrontal and anterior cortices has to be made with great caution; the results reported in the present study should be seen as preliminary and they await replication in further MRI investigations free from potentially confounding effects of the use of variable scanners and data acquisition protocols.

Some other limitations of this study should also be considered. The investigation of the impact of other CR proxies on the association between aging and brain volume reduction were not the scope of this study. In this scenario, we cannot rule out the possibility that differences in socioeconomic status, occupation, intellectual abilities and physical activities could at least partially interfere in the brain aging processes [60,61]. One relevant aspect is that the age of low-education group was only slightly, but significantly higher than the high education group and this could have an impact in the analysis of the association between rGM volumes and aging according to the level of schooling of the participants. One other possible limitation is the unbalanced size of the samples of high and low education. According to the Brazilian Institute for Geography and Statistics (2014), 26.5% of elders with more than 60 years old are illiterate, which partially justify our larger sample with low educational attainment. Our sample of low education subjects was mainly composed of elders from the SPAH cohort (Study 1) who mostly born in Brazilian rural areas and represent those who survived very high infant mortality and endured much socio-economic adversity throughout their lives [62]. This scenario implies that these people grew up in particularly deprived conditions, and the impact of this physical and cultural deprivation may also have an effect on brain development and could indirectly influence our results. Finally, two persons with the same level of education could have had devoted different amounts of time to school. This imposes a possible bias to the measurement of educational attainment of our sample. Future studies, especially with larger samples of illiterate participants are therefore necessary to corroborate our findings. Besides, future longitudinal MRI studies following samples of low education subjects are warranted to directly attest if these people with very low educational level are more vulnerable to brain degenerative changes than subjects with longer educational attainment.

## Conclusion

In conclusion, our findings provide preliminary evidence that educational attainment may have a subtle protective effect on age-related brain changes in healthy elderly subjects. Brain regions that are critical to inhibitory control processes and other cognitive operations, such as the anterior cingulate cortex, might be particularly sensitive to such protective effects, which may derive directly from previous cognitive stimulating activities exercised at school per se or indirectly through the development and maintenance of education-related cognitive resources throughout life.

## Supporting Information

S1 File **Scanning sequences used in each of the three studies (Table A).** Matrix size (in voxels; a x b x c; c = number of slices); voxel size (in mm; a x b x c; a x b = in plane resolution; c = slice thickness); N = number of participants; TR: repetition time; TE: echo time; FA = flip angle. **Demographic characteristics of the participants from each previous study that contributed MRI data (Table B).** ^a^ Study 1 ≠ Study 3; ^b^ Study 1 ≠ Study 2. **Distribution of participants with high and low education across age ranges (<70 and > = 70 years-old) (Table C).**
(DOCX)
