# Supplementary material for: Relationship between Brain Age-Related Reduction in Gray Matter and Educational Attainment
Source: PLoS One. 2015 Oct 16;10(10):e0140945. doi: 10.1371/journal.pone.0140945 (PMC4608774; doi:10.1371/journal.pone.0140945)
Supplement: S1 File — Scanning sequences used in each of the three studies (Table A). Matrix size (in voxels; a x b x c; c = number of slices); voxel size (in mm; a x b x c; a x b = in plane resolution; c = slice thickness); N = number of participants; TR: repetition time; TE: echo time; FA = flip angle. Demographic characteristics of the participants from each previous study that contributed MRI data (Table B). a Study 1 ≠ Study 3; b Study 1 ≠ Study 2. Distribution of participants with high and low education across age ranges (<70 and > = 70 years-old) (Table C). (DOCX) [file pone.0140945.s001.docx]

**S1 File Table A**. Scanning sequences used in each of the three studies.

|  |  | **Type of 1.5T MRI Scan** | |  |  |  |  |  |  |  |
| --- | --- | --- | --- | --- | --- | --- | --- | --- | --- | --- |
| **Study** | **N** | **Vendor** | **Model** | **Sequence** | **TR (ms)** | **TE (ms)** | **FA (^o^)** | **Orientation** | **Matrix Size** | **Voxel Size** |
| Study 1 | 156 | General Eletric | Signa | SPGR | 21.7 | 5.2 | 20 | Coronal | 256 x 192 x 220 | 1 x 1 x 1.50 |
| Study 2 | 14 | General Eletric | Signa | FSGE | 6.5 | 1.49 | 15 | Coronal | 256 x 256 x 124 | 0.94 x 0.94 x 1.20 |
| Study 3 | 18 | Phillips | Gyroscan S15-ACS | FFE T1 | 30 | 9 | 30 | Coronal | 256 x 256 x 240 | 0.94 x 0.94 x 1.20 |

Note: Matrix size (in voxels; a x b x c; c=number of slices); voxel size (in mm; a x b x c; a x b=in plane resolution; c=slice thickness); N=number of participants; TR: repetition time; TE: echo time; FA=flip angle.

**S1 File Table B.** Demographic characteristics of the participants from each previous study that contributed MRI data

|  | **Study 1 (n=156)** | **Study 2 (n=14)** | **Study 3 (n=18)** | **Statistical Test** | |
| --- | --- | --- | --- | --- | --- |
|  | **Mean (SD)** | **Mean (SD)** | **Mean (SD)** | **F** | **p** |
| Age (years) | 70.33 (2.33) | 69.43 (5.92) | 70.33 (7.66) | 0.42 | 0.656 |
| Education level (years) | 4.71 (3.68) | 7.14 (4.67) | 9.56 (4.96) | 14.10 | <0.001^a^ |
|  | **N (%)** | **N (%)** | **N (%)** | **Fisher’s exact test** | **p** |
| Male | 78 (50%) | 8 (57.1%) | 4 (22.2%) | 5.51 | 0.064 |
| Low Education | 112 (71.8%) | 5 (35.7%) | 5 (27.8%) | 19.38 | <0.001^a,b^ |

Note: ^a^ Study 1 ≠ Study 3; ^b^ Study 1 ≠ Study 2

**S1 File Table C. Distribution of participants with high and low education across age ranges (<70 and >=70 years-old)**

|  | **<70 years-old** | **>=70 years-old** | **X^2^ or Fishers’ exact test** | **p** |
| --- | --- | --- | --- | --- |
| **Study 1** | | | | |
| Low education | 43 | 69 | 0.654 | 0.419 |
| High education | 20 | 24 |  |  |
| **Study 2** | | | | |
| Low education | 3 | 2 |  | 1.00 |
| High education | 6 | 3 |  |  |
| **Study 3** | | | | |
| Low education | 0 | 5 |  | 0.036* |
| High education | 8 | 5 |  |  |

Note: * Refers to statistical difference.
